# Bone marrow mesenchymal stem cell aggregate: an optimal cell therapy for full-layer cutaneous wound vascularization and regeneration

**DOI:** 10.1038/srep17036

**Published:** 2015-11-23

**Authors:** Yulin An, Wei wei, Huan Jing, Leiguo Ming, Shiyu Liu, Yan Jin

**Affiliations:** 1 State Key Laboratory of Military Stomatology, Center of Tissue Engineering, School of Stomatology, The Fourth Military Medical University, No. 145 Changlexi Road, Xi’an, Shaanxi 710032, China; 2 Research and Development Center for Tissue Engineering, Fourth Military Medical University, No. 145 Changlexi Road, Xi’an, Shaanxi 710032, China; 3 Zhen Jiang Entry-Exit Inspection And Quarantine Bureau, No. 84 Dongwu Road, Zhen Jiang, Jiang Su 212000, China; 4 State Key Laboratory of Military Stomatology, Department of Periodontology, School of Stomatology, The Fourth Military Medical University, No. 145 Changlexi Road, Xi’an 710032, China

## Abstract

Cutaneous wounds are among the most common soft tissue injuries. Wounds involving dermis suffer more from outside influence and higher risk of chronic inflammation. Therefore the appearance and function restoration has become an imperative in tissue engineering research. In this study, cell-aggregates constructed with green fluorescent protein-expressing (GFP^+^) rat bone marrow mesenchymal stem cells (BMMSCs) were applied to rat acute full-layer cutaneous wound model to confirm its pro-regeneration ability and compare its regenerative efficacy with the currently thriving subcutaneous and intravenous stem cell administration strategy, with a view to sensing the advantages, disadvantages and the mechanism behind. According to results, cell-aggregates cultured *in vitro* enjoyed higher expression of several pro-healing genes than adherent cultured cells. Animal experiments showed better vascularization along with more regular dermal collagen deposition for cell-aggregate transplanted models. Immunofluorescence staining on inflammatory cells indicated a shorter inflammatory phase for cell-aggregate group, which was backed up by further RT-PCR. The *in situ* immunofluorescence staining manifested a higher GFP^+^-cell engraftment for cell-aggregate transplanted models versus cell administered ones. Thus it is safe to say the BMMSCs aggregate could bring superior cutaneous regeneration for full layer cutaneous wound to BMMSCs administration, both intravenous and subcutaneous.

Skin acts as the protective layer of human body and therefore becomes the most vulnerable part of human. Mechanical scraping and burning or ambustion are the most common acute traumas to skin. While skin wounds upon epidermis tend to heal in a few days, deeper wounds involving dermal damage requires longer healing cycle, sometimes with hampered the function restoration and higher risk of chronic inflammation or further infection^1^,^2^.

The full-thickness cutaneous wound typically causes damage to many structures and cell lineages, whose healing and regeneration begins right after wounding^3^. The healing and regeneration process were orchestrated by a number of growth factors such as VEGF and TGF-β, of which, the mesenchymal stem cell (MSC) had been considered as a good source, and a promising treatment to such wounds^4^,^5^,^6^,^7^.

Currently, MSC mediated therapies were applied to many injury treatments wherein system and topical cell administration had been frequently adopted^8^,^9^,^10^,^11^. Such administration therapies, counting on enzyme-digested single cell suspension *in vitro* and stem cell migration to wound area *in vivo*, had always suffered from unguaranteed cell quantity and quality and limited the exertion of MSC functions^12^. The cell-aggregate strategy had been introduced to many tissue damage treatments such as bone fracture and cardiac damage^13^,^14^,^15^. Without any enzyme-digestion, the cell-aggregate magnified the advantage of matrix as scaffold to bring better cell vitality and manageable size to wound area transplantation^16^.

We aimed to combine such advantages with GFP^+^BMMSC immune-regulation and pro-healing properties to achieve shorter inflammatory phase and optimal dermal regeneration. In this study, GFP^+^BMMSC were acquired and cultured in medium containing dexamethasone and ascorbic acid phosphate to form cell aggregate that could be scraped off mechanically, and were then transplanted onto rat acute cutaneous wound models without any scaffold. Same number of GFP^+^BMMSCwere administered topically around the wound and intravenously through tail vein on homogeneous models respectively. Wound bed samples at different time points were collected. Morphological, histological and molecular evidence were analyzed to compare healing and regeneration results of three transplantation approaches and to deduce the probable mechanism.

## Results

### Construction of GFP^+^BMMSC aggregate and its characteristics

**Figure 1:**
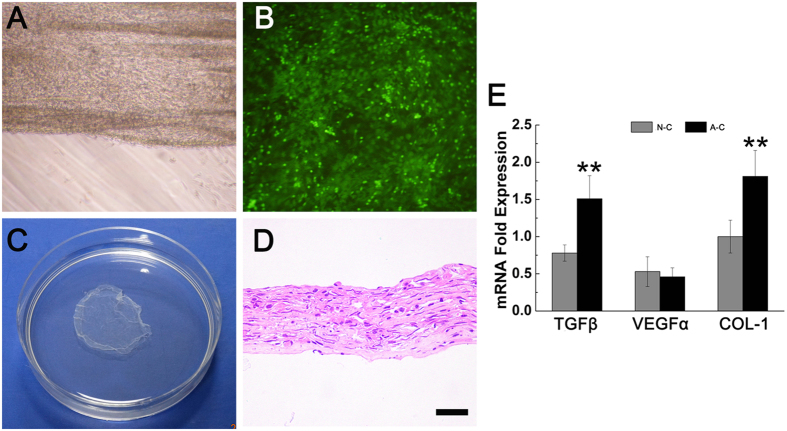
GFP^+^BMMSC aggregate construction and expression characteristic. (**A**) The rim of aggregate curled a little on dish bottom. (**B**) GFP^+^BMMSCs in the aggregate gave green fluorescence under 509 nm excitation light. (**C**) The whole aggregate was scratched off the dish. (**D**) HE staining revealed a certain thickness of the aggregate with cells in it (Bar = 20 nm). (**E**) RT-PCR showed that BMMSC aggregate presented significantly higher expression of TGF-β and collagen I but had a similar VEGFα expression with normal cultured cells. (N-C: normal cultured cells; A-C: Aggregate cells; **p < 0.05 is considered statistically different.)

The primary GFP^+^BMMSC were isolated and cultured *in vitro*. Multi-differentiation test demonstrated their osteogenesis and adiposgenesis potential (Supplementary Fig. 1A,B). They exhibited fibroblast like or long fusiform shape and gave green fluorescence under fluorescence microscope (509 nm) (Supplementary Fig. 1C). Flow cytometry test demonstrated positive expression of mesenchymal stem cell markers CD29, CD90 and Sca-1 and negative expression of the hematopoietic markers CD45, CD34 and CD14 (Supplementary Fig. 1D.) BMMSCs were cultured *in vitro* for 12 days in the media containing Vitamin C and dexamethasone, and an ivory membrane could be seen at bottom of dish, with its rim curling a little. This cell-aggregate, where cells grew overlapped (Fig. 1A,B), could be detached mechanically from the bottom and remain unfolded in PBS (Fig. 1C). HE staining of cell-aggregate showed a membrane structure made of collagen, with 53.5 ± 4.0 μm thickness and MSCs buried inside (Fig. 1D). 10 GFP^+^BMMSC aggregates constructed at three different batches were digested into single cell suspension. Electronic cytometer revealed an average number of (1.35 ± 0.07)×10^6^ cell for one aggregate. In accordance to this number, single cell suspension containing 1.35 × 10^6^ GFP^+^BMMSC was prepared in physiological saline for administration.

The pro-healing genes detected *in vitro* included collagen type I(COL I), vascular endothelial growth factor α (VEGF-α) and transforming growth factor β (TGF-β) (Fig. 1E). As shown in RT-PCR results, BMMSC aggregate expressed significantly higher collagen type I(*p* = 0.011), which was confirmed by the HE staining above. Previous studies had proved several times that collagen type I was of great importance to MSCs niche improvement and vitality^17^,^18^. TGF-β expression in BMMSC aggregate was also much higher than BMMSC in α-MEM (*p* = 0.014) while VEGF-α manifested a similar expression level between them (*p* = 0.201). Taken together, after inducing for 12 days, GFP^+^BMMSC could form a cell-aggregate with certain thickness and expressed higher level of TGF-β and certain level of VEGF-α, thus holding certain capacity for angiogenesis and promoting fibroblast proliferation.

### Wound size and collagen deposition of wound bed skin

**Figure 2:**
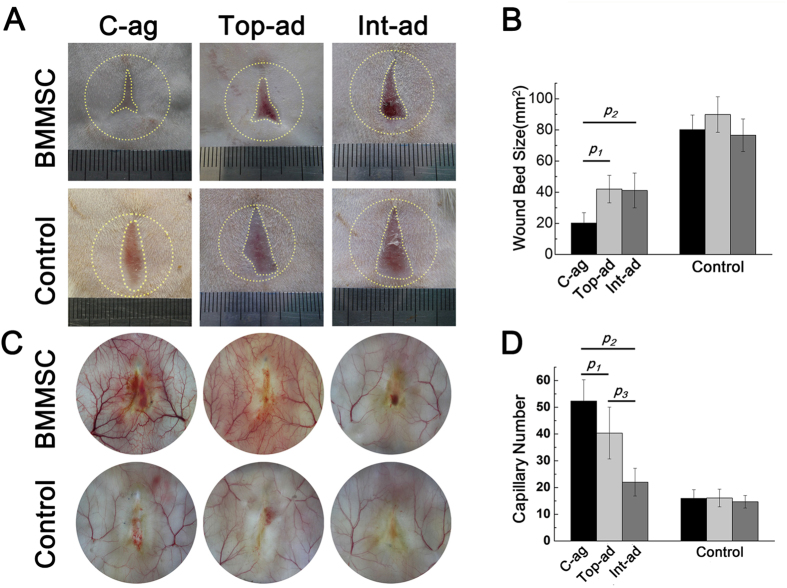
Wound bed size and vascularization state. (**A,B**) Wound bed size and vascularization state in BMMSC-transplanted rats with each control at 4-week post-operation. Yellow dashed circle outside and the dashed line inside showed the original wound size and the left wound bed respectively; (**C**) Quantification of wound bed size revealed that rats of C-ag group had the smallest wound bed left at 4W. *p*_*1*_ = 0.000, *p*_*2*_ = 0.001, *n* = 14; (**D**) Quantification of capillary number revealed that C-ag group models enjoyed the highest capillary density followed by models of Top-ad group and Int-ad group (*p*_*1*_ = 0.001, *p*_*2*_ = 0.000, *p*_*3*_ = 0.000, *n* = 15).

**Figure 3:**
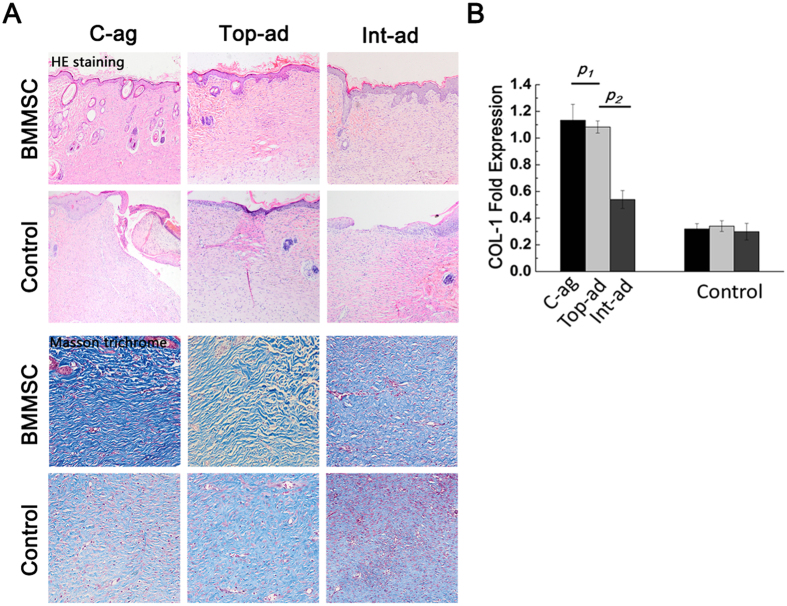
Epithelialization and collagen deposition state. (**A**) HE staining (top, 10×) of 4-week samples showed epithelialization in three BMMSC transplanted groups while not in all their controls. Masson trichrome staining (bottom, 20×) of dermal layer showed a superior collagen deposition with certain direction and thicker bundle for C-ag group and Top-ad group to that of Int-ad group, while the collagen disposition of control groups samples was short without certain direction; (**B**) RT-PCR confirmed that the samples of C-ag group and the Top-ad group presented the highest collagen I expression among groups and followed by Int-ad group (*p*_*1*_ > 0.05, *p*_*2*_ < 0.05).

All 96 chosen host rats survived until scarification and 6 of them were rolled out for acute infection. As demonstrated by Fig. 2A, 4-week post-surgery, all animals had achieved wound closure. Wound area size of BMMSC-aggregate (C-ag) group animals was (6.43 ± 2.28) % of the original size, and the number was (13.12 ± 3.01) % and (13.10 ± 3.55) % in topical-administration (Top-ad) and intravenous-administration (Int-ad) group respectively. Statistical analysis further confirmed the significance (Fig. 2B, *p*_*1*_ = 0.000, *p*_*2*_ = 0.001). The size of three BMMSC transplanted groups were significantly smaller than each of their controls (Fig. 2C, *p*_*c*_ = 0.000, *p*_*top*_ = 0.001, *p*_*int*_ = 0.002). HE staining of 4-week samples showed epithelialization in three BMMSC transplanted groups while not in their controls. Masson trichrome staining of dermal layer showed a superior collagen deposition with certain direction and thicker bundle for C-ag group and Top-ad group versus that of Int-ad group (Fig. 3A), while the collagen deposition of control groups samples was short without certain direction. This advance was further backed up by the higher COL-1 expression in the first two groups which had no significant difference between them (Fig. 3B, *p*_*1*_ > 0.05, *p*_*2*_ < 0.05).

### Vascularization of wound bed skin

Vascularization was of essence to wound healing and tissue repairing. In this study, vascularization level varied among groups. At 4-week post-surgery, blood vessels and capillary net growing into the center of wound bed were much more intense in C-ag group than in the two cell administration groups (Fig. 2C). Capillaries within the original wound size (2 cm diameter circle) were quantified by software. Statistical results showed that C-ag group models enjoyed the highest capillary density (52.3 ± 7.96, *n* = 15) followed by models of Top-ad group (40.4 ± 9.68, *n* = 16) and Int-ad group (22 ± 5.18, *n* = 15), difference being significant (Fig. 2D, *p*_*1*_ = 0.011, *p*_*2*_ = 0.000, *p*_*3*_ = 0.000). Generally, BMMSCs transplantation group enjoyed higher capillary density than their controls (Fig. 2D, *p*_*c*_ = 0.002, *p*_*top*_ = 0.004, *p*_*int*_ = 0.017). The optimized vascularization in wound bed tissue has been reported to be accompanied with better healing results either in morphological or in histological term^19^,^20^,^21^, which in this study was backed up by the results above. Therefore the better vascularization state brought by cell aggregate transplantation had posed an advantage over MSCs administration in facilitating healing and regeneration.

### Inflammation infiltration and inflammatory factors expression

To explore details of skin healing among different groups, inflammatory cytokines expression in wound bed tissue was also detected.

**Figure 4:**
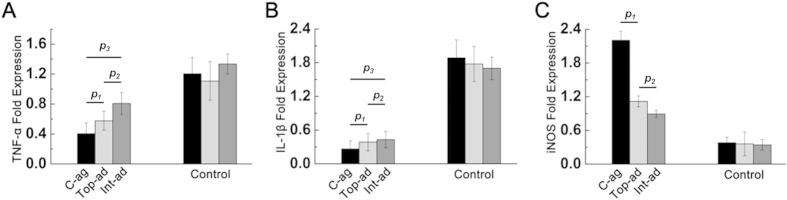
Inflammatory state. RT-PCR showed the expression profile of inflammatory cytokines TNF-α (**A**, *p*_*1*_ < 0.05, *p*_*2*_ < 0.05, *p*_*3*_ < 0.01) and IL-1β (**B**, *p*_*1*_ > 0.05, *p*_*2*_ > 0.05, *p*_*3*_ < 0.05) and immune-regulating gene iNOS (**C**, *p*_*1*_ < 0.05, *p*_*2*_ < 0.05). Wound bed tissues of C-ag and Top-ad group expressed lower level of TNF-α and IL-1β, which were significantly higher in Control groups. Tissue of C-ag group expressed highest level of iNOS among groups.

At 2-week post-operation, BMMSC transplanted groups exhibited faster inflammation regression than their controls, with C-ag group specimen bearing the lowest level of TNF-α and IL-1β (Fig. 4A, *p*_*1*_ < 0.05, *p*_*2*_ < 0.05, *p*_*3*_ < 0.01; Fig. 4B, *p*_*1*_ > 0.05, *p*_*2*_ > 0.05, *p*_*3*_ < 0.05). On top of that, the highest level of inducible nitric oxide synthase (iNOS) expression was also detected in C-ag group out of three cell transplanted groups, whereas the other two groups had no statistical difference (Fig. 4C, *p*_*1*_ < 0.05, *p*_*2*_ < 0.05). INOS level was almost the same in controls, being significantly lower than BMMSC transplanted specimen.

**Figure 5:**
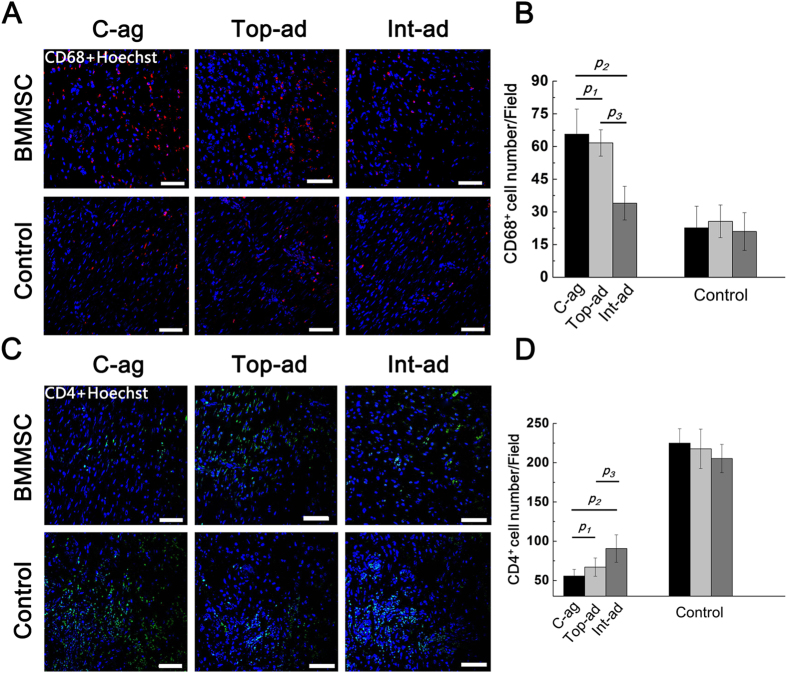
(**A**) Immunofluorescence staining on CD68^+^ macrophage on wound bed samples at 2W (counterstained with Hoechst 33342) (Bar = 50 nm); (**B**) Quantification of CD68^+^ cell among groups. C-ag group samples bore the highest macrophage infiltration at 2W followed by that of Top-ad and Int-ad group. *p*_*1*_ = 0.071, *p*_*2*_ = 0.000, *p*_*3*_ = 0.000, *n* = 6; (**C**) Immunofluorescence staining on CD4^+^ T cell on wound bed samples at 2W (counterstained with Hoechst 33342) (Bar = 50 nm); (**D**) Quantification of CD4^+^ T cell among groups. C-ag group samples and Top-ad group samples presented the lowest CD4^+^ T cell infiltration at 2W followed by that of Int-ad group. *p*_*1*_ = 0.052, *p*_*2*_ = 0.000, *p*_*3*_ = 0.011, *n* = 6.

Immune-fluorescence evidence revealed the relations between inflammation and BMMSCs transplantation from another angle. Macrophage, marked by CD68, was considered as the main force behind therapeutic inflammation and granulation formation^22^,^23^,^24^. Therefore, macrophage infiltration set the premise in the early stage of wound healing process. As shown in Fig. 5A, CD68^+^ macrophages infiltration exhibited different state at 2W post-operation among groups. Generally, macrophages in BMMSCs transplantation groups posed a contrast with their controls (Fig. 5B). Macrophages detected in C-ag and Top-ad group were significantly more than that in Int-ad group, with no significance between them (Fig. 5B, *p*_*1*_ = 0.071, *p*_*2*_ = 0.000, *p*_*3*_ = 0.000, *n* = 6).

T cells, as the main force of immune defense besides inherent immune cells, played an vital role in secreting cytokines and regulating inflammation during healing process^25^. At 2W post-operation, CD4^+^ T cell infiltration varied among groups (Fig. 5C). BMMSCs transplantation groups presented a striking contrast of CD4^+^ T cell infiltration compared with their controls. While among them, specimen of C-ag and Top-ad models enjoyed the lowest infiltration followed by Int-ad group (Fig. 5D, *p*_*1*_ = 0.052, *p*_*2*_ = 0.000, *p*_*3*_ = 0.011, *n* = 6). Such results had corresponded with the previous PCR results of iNOS from wound bed tissue.

**Figure 6:**
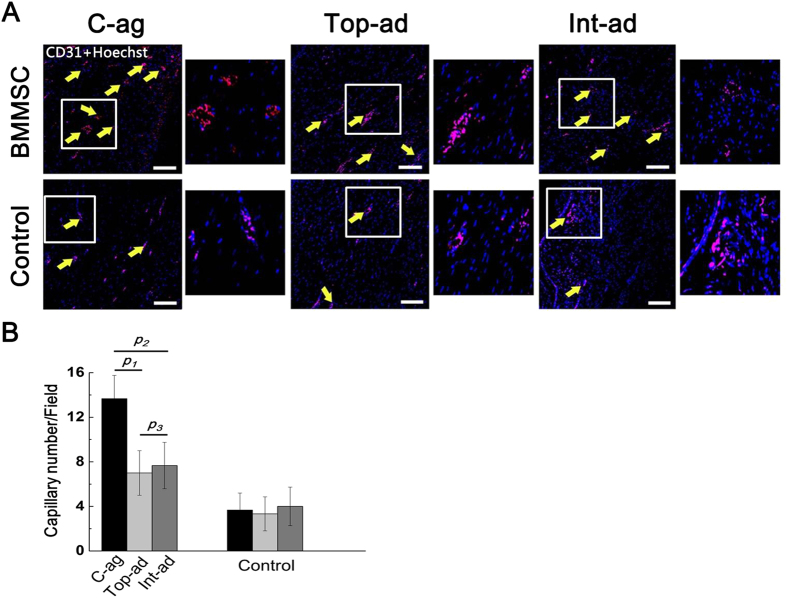
(**A**) Immunofluorescence staining on CD31^+^ endothelial cell on wound bed samples at 4W (counterstained with Hoechst 33342) (Bar = 50 nm). Yellow arrow pointed out the location of capillaries; small pictures on the right showed the amplification of frame area in the left; (**B**) Quantification of CD31^+^ cell among groups. Capillaries as marked by CD31^+^ endothelial cell in C-ag wound bed tissue were significantly more than that of Top-ad and Int-ad group. *p*_*1*_ = 0.000, *p*_*2*_ = 0.000, *p*_*3*_ = 0.804, *n* = 6.

To evaluate the vasculature reconstruction and the inflammatory state at 4W post-operation, we conducted immunofluorescence on CD31 and CD45 to further confirm morphological evidence. According to results, number of capillary as marked by CD31^+^ endothelial cell in cell-aggregate transplanted wound bed tissue was significantly higher than that of the cell administration groups, while all controls bore much less capillaries than their BMMSCs transplanted counterparts (Fig. 6A,B, *p*_*1*_ = 0.000, *p*_*2*_ = 0.000, *p*_*3*_ = 0.804, *n* = 6).

**Figure 7:**
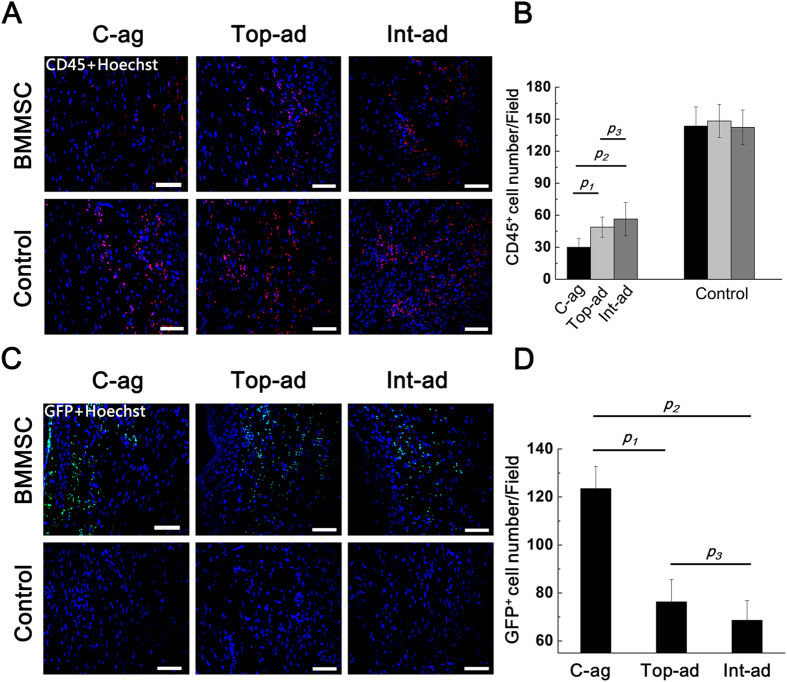
(**A**) Immunofluorescence staining on CD45^+^ lymphocytes on wound bed samples at 4W (counterstained with Hoechst 33342) (Bar = 50); (**B**) Quantification of CD45^+^ cell among groups. CD45^+^ cell infiltration in Top-ad and Int-ad wound bed tissue was heavier than that that in C-ag ones. *p*_*1*_ = 0.003, *p*_*2*_ = 0.000, *p*_*3*_ = 0.405, *n* = 6; (**C**) Immunofluorescence staining on GFP^+^ cell on wound bed samples at 4W (counterstained with Hoechst 33342) (Bar = 50 nm); (**D**) Quantification of GFP^+^ cell among groups indicated better engraftment for C-ag group than the other two cell transplanted groups, difference being significant. *p*_*1*_ = 0.001, *p*_*2*_ = 0.000, *p*_*3*_ = 0.135, *n* = 6.

Being the surface marker of bone marrow derived lymphocytes^26^,^27^, CD45^+^ cell infiltration indicated the ongoing of inflammation on wound bed^28^,^29^. Here, immunofluorescence staining on CD45^+^ cells presented a converse tendency to CD31. As shown in Fig. 7A,B, at 4W post-operation, CD45^+^ cell infiltration in BMMSC topical administration and intravenous administration wound bed tissue was still heavier than that in cell-aggregate transplanted ones, difference being significant (*p*_*1*_ = 0.000, *p*_*2*_ = 0.000, *p*_*3*_ = 0.141, *n* = 6). The infiltration state of MSCs transplantation groups was much lighter than that of control groups.

As the essential indicator of transplantation efficacy, GFP^+^BMMSCs engraftment was also examined at 4-week post-operation. According to Fig. 7C, GFP^+^ cells can be detected at all three BMMSCs transplanting groups. Quantification of immunofluorescence indicated the better engraftment for C-ag group than the other two groups, difference being significant (Fig. 7D, *p*_*1*_ = 0.001, *p*_*2*_ = 0.000, *p*_*3*_ = 0.135, *n* = 6).

## Discussion

Skin is the biggest organ of human body, and its healing and regeneration after acute wound becomes a hot spot in regenerative medicine recently. BMMSC-based therapies are currently thriving in facilitating cutaneous wound healing^8^,^9^,^11^,^30^. In those therapies, intravenous and topical administration are frequently adopted as BMMSC transplanting strategy, but their mechanism remains controversial and the unsteady results as well as the low cell engraftment have left much room for improvement^31^.

Cell-aggregate formed by mesenchymal stem cells has been successfully adopted in promoting wound healing of several organs, in which skin are not included^15^,^32^,^33^.

In this study, three BMMSCs transplantation strategies were applied on acute full-layer skin wound models on SD-rats. According to results, the cell-aggregate transplantation models yielded faster re-epithelialization and optimized dermal collagen deposition than models of cell administration groups. Such an advantage endowed better mechanical property and ductility to neo-skin tissue and also reduced the risk of re-dilaceration and infection in C-ag group rats.

Studies have been concentrating on MSCs-mediating neovascularization when it comes to MSCs therapies. It is reported that MSC neovascularization-promoting capability relied largely on its paracrine effects, or rather cytokines such as VEGFs and Ang-1^6^,^34^,^35^. Our results exhibited greater capillary density for C-ag models at 4-week post-operation. Despite BMMSC aggregate secreted similar amount of VEGFα to MEM-cultured BMMSCs *in vitro*, its promoting effect was not bothered *in vivo* because cell-aggregate transplantation enjoyed much higher stem cell engraftment as manifested by GFP-immunohistochemistry. The better GFP^+^BMMSC engraftment was the critical advance brought by cell-aggregate transplantation because it underpinned the pro-healing functions of BMMSC. This advance could be even more highlighted in the elders whose vasculature and regeneration ability tend to decline along aging process^1^,^21^,^36^. However, it requires another confirmation on old animal models.

Besides collagen deposition and vascularization, inflammation among different group was also compared. It was the consensus that BMMSC’s immune regulation ability could exert positive influence on inflammation process, preventing it from chronic inflammation^37^,^38^. Such a function requires the participation of particular immune repressive protein such as iNOS, and more importantly, it relies on sufficient BMMSC engraftment on wound bed^31^,^39^,^40^. Our results witnessed lighter inflammation level for C-ag models at 2W post-operation accompanied by more recruited macrophages. The later indicated an earlier arrival of reconstruction procedure than Top-ad and Int-ad models. In this study, the higher iNOS expression in C-ag models was accompanied by less CD4^+^ T cell infiltration at 2W post-operation, reflecting a faster inflammation regression. This trend persisted till 4W post-operation as confirmed by less CD45^+^ cell infiltration. However, it should be noticed that the inflammation difference wasn’t manifested upon cell transplantation because the expression of inflammatory cytokines in each group barely presented any variation at 1-week post-operation (Supplementary Fig. 3).

Previous studies had proved BMMSC paracrine effects in promoting wound healing and facilitating regeneration^7^,^41^,^42^. For example, it was reported that TGF-β1 could shift keratinocyte integrin toward a more migratory phenotype or enhance their proliferation to facilitate re-epithelialization^43^,^44^,^45^. It also augmented macrophage mediated tissue debridement and prepared the wound for granulation tissue formation^46^. As to vascularization, VEGFα was considered essential in endothelial cell migration^47^,^48^ and proliferation^49^,^50^. TGF-β and VEGFα expression could be enhanced by each other *in vivo*^51^,^52^. Immune-regulation was another important aspect of BMMSC effect. The mechanism had been intensively studied^40^,^53^,^54^,^55^. Many MSCs derived factors had been identified that could reduce immune cell inflammation, including TGF-β, IL-6, IL-10, iNOS, Gal-1, HLA-G and so on^56^,^57^,^58^,^59^. MSCs engrafted at the wounded tissue were efficient in producing these soluble agents to alter the course of the local inflammatory response^7^. Previous studies had proved that this paracrine-based pro-regeneration effects of BMMSCs required sufficient engraftment to local microenvironment^18^,^60^,^61^,^62^,^63^. Therefore, better engraftment laid the foundation of optimal healing^19^,^64^.

Evidence here showed that cell-aggregate transplanted rat models enjoyed better engraftment in neo-skin tissue, which corresponded with previous studied on other organs^13^,^15^. This result reflected the advantage of aggregate of providing more favorable microenvironment for MSCs survival. And this could be the pivotal advantage in cell-aggregate application as a biomaterial.

In this study, we proved the efficiency of BMMSC aggregate in facilitating full-layer cutaneous wound healing and regeneration. Its transplantation upon wound model enjoyed better cell engraftment compared with either intravenous or topical BMMSC administration, but the mechanism behind needed further study.

## Conclusion

BMMSC aggregate transplantation presented better neo-vascularization and more regular collagen deposition compared to either intravenous or topical equal-volume BMMSC administration on acute cutaneous wound model, thus bringing higher healing and regenerative efficacy. The mechanism behind could be the better regulated inflammatory process owing to the higher BMMSC engraftment brought by cell-aggregate. BMMSC aggregate transplantation might provide a new sight into acute cutaneous wound regeneration, experimentally and clinically.

## Materials and Methods

### Ethics statement

All experimental protocols were approved by the Fourth Military Medical University. All animal experiments conducted in this research were performed in accordance with the guidelines of the Fourth Military Medical University Intramural Animal Use and Care Committee and met the NIH guidelines for the care and use of laboratory animals.

### Experimental animals

20 green GFP^+^ Sprague-Dawley rats (GFP^+^-rats) bought from Vital-river Company(Peking, China) were used as donors of GFP^+^BMMSCs. 2-week old GFP^+^-rats were sacrificed for GFP^+^-BMMSCs isolation and culture. 96 female SD rats were obtained from the Laboratory Animal Research Centre of the Fourth Military Medical University.

200 g–220 g healthy female SD rat were adopted for making acute cutaneous wound model. All 96 chosen host rats were randomly divided into 6 groups, the GFP^+^BMMSC aggregate transplantation group (C-ag group), intravenous administration group (Int-ad group) and topical administration group (Top-ad group), each with a control group(Con group). All GFP^+^-rats were normally raised for one week under pathogen-free conditions (22 °C, 12-h light/12-h dark cycles and 50–55% humidity) for acclimation before sacrificed. Host rats were raised under the same condition with proper density and free access to food and water before or after the surgery. All surgical procedures were performed under general anesthesia with intra-peritoneal injection of 1% pentobarbital under sterilized situation.

## Isolation and Identification

The isolation and primary culture procedure of GFP^+^BMMSC has been previously reported^15^,^16^. In brief, freshly harvested BMMSCs were seeded in 100 mm culture dishes with α-MEM (Gibco BRL, Gaithersburg, MD, USA) supplemented with 10% FBS, 2 mM-glutamine (Invitrogen, Carlsbad, CA, USA), 100 U/ml penicillin, and 100 mg/ml streptomycin (Invitrogen, Carlsbad, CA, USA) changed every alternative day. 3^rd^ passage GFP^+^BMMSCs were adopted for flow cytometry analysis and stem cell characteristics identification. The procedure of flow cytometry analysis was like previously reported^65^. In brief, surface marker CD90 (PE), SCA-1 (FITC), CD29 (PE), CD45 (PE), CD34 (FITC) and CD14 (PE) (BD Bioscience, San Jose, CA, USA) were detected with a Beckman Coulter EpicsXL cytometer (Beckman Coulter, Fullerton, CA, USA). Multi-linage differentiation of MSC was conducted as previously reported^64^. Briefly, osteogenic differentiation was induced by α-MEM containing 0.1 μM dexamethazone, 50 μM ascorbic acid, and 10 mM glycerophosphate; adipogenic differentiation was induced by α-MEM with 0.5 μM dexamethazone, 0.5 mM 3-isobutyl-1-methylxanthine, and 0.1 mM indo-methacine.

### Cell-aggregate and single cell suspension preparation

GFP^+^BMMSCs of 3^rd^ passage were used to construct GFP^+^BMMSC aggregate. Cells were seeded at the density of 5 × 10^4^/cm^2^ on 50 mm-diameter culture dishes at 37 °C in a humidified atmosphere containing 5% CO_2_. Cells reaching 90% confluence would receive inducing media containing 100 mg/mL Vitamin C and 10 nM dexamethasone until a white membrane structure could be observed. Culture was continued for 14 days to make aggregate thicker. 10 completed GFP^+^BMMSC aggregates constructed in three different batches were digested with 0.25% trypsin/1 mM EDTA into single cell suspension which were then enumerated by electronic cytometer for the average cell number in one aggregate. Then the same number of 3^rd^ passage GFP^+^BMMSCs was prepared into single cell suspension for *in vivo* administration experiments.

### Collagen and cytokines expression profile of scattered cell and cell aggregates

To assess the pro-healing capacity *in vitro*, we examined expression of collagen I and several other cytokines that were critical to wound healing and regeneration by GFP^+^BMMSCs aggregate and scattered cells. Total RNA was extracted with Trizol Reagent (Invitrogen) from ripe GFP^+^BMMSCs aggregates and GFP^+^BMMSCs that was normally cultured for the same time and reversely transcribed into cDNA using a synthesis kit (TaKaRa, Japan). RNA extraction procedure was like previously reported^66^. Target genes and primers were presented in Table 1, β-actin being internal control. Normalization and fold changes were calculated by ΔΔCt method.

### Acute rat cutaneous wound model and transplantation

All chosen host SD rats were individually anesthetized by intra-peritoneal injection of 1% pentobarbital sodium and their dorsum surface was shaved first by an electronic clipper and then a surgical blade to ensure no hair remaining. A 2 cm diameter full thickness cutaneous wound was carefully made on the top half of the back right across the midline of dorsum by an ophthalmic scissors after a punch biopsy tool outlining the pattern (Supplementary Fig. 2). To ensure the homogenicity of wound models, subcutaneous panniculus carnosus was cleaned out and all wound models were subjected to transplantation within 30 seconds under sterilized situation.

For C-ag group, ripe GFP^+^BMMSC aggregate, washed three times previously, was carefully scratched off the culture dish with a probe, and paved as a cover on the wound bed. Three layers of ethylene oxide sterilized SIS membrane (3 cm diameter), whose biocompatibility had been proved previously^20^, were then covered on the cell aggregate to separate it from upper dressings. The SIS membrane was first infiltrated in physiological saline to avoid air bubble. On top of that a monolayer air permeating wound dressing was applied (Tegaderm film1624, 3 M, USA), which covered only shaved peripheral skin and was then sutured up. The control group rats were subjected to the same procedure without cell aggregates.

200 μl single cell suspension containing the same number of 3^rd^ passage GFP^+^-BMMSCs as cell-aggregate was prepared for administration. For topical administration (Top-ad), 200 μl cell suspension was administered subcutaneously at 4 sites around the wound bed via a syringe, with 3 mm to the rim. Its control group was administered with same volume of physiological saline. For intravenous administration (Int-ad), 200 μl cell suspension was administered though caudal vein with control group rats receiving the same volume of physiological saline. The procedure after cell administration was the same to C-ag group.

### Wound healing assessment

To evaluate the healing efficacy of host rats, size of wound bed at 4-week post-operation were imaged by digital camera and quantified by Image J image analysis software (NIH image software). The percentage of wound closure was calculated as follows: (area of original wound–area of actual wound)/area of original wound × 100%. Rats of 6 groups were sacrificed at 4W post-surgery and approximately 5 × 5 cm full-thickness cutaneous biopsies of the wound bed with surrounding skin were harvested and placed on the bottom of polystyrene cell culture dish with inner face down. Subsequently, the vascular infiltration state of each specimen was captured by digital camera with a standard incandescent illumination directed behind. The neo-capillaries in wound bed area were quantified.

### Histological analysis and immunofluorescence analysis

Wound bed tissues of each group were harvested at 1, 2 and 4 weeks post-surgery and specimen of 2W (wound bed at 2 weeks) and 4W (wound bed at 4 weeks) were divided into two parts along their central axis. One half was fixed with 4% paraformaldehyde and routinely processed to 5 μm-thick paraffin-embedded sections. The other half was used for RNA extraction.

Deparaffinized sections of 4W tissue specimen were subjected to Masson staining for collagen accumulation state with a staining kit^67^. HE staining was conducted as previously described^68^. 5 μm thick paraffin slices were subjected to staining. 6 pictures of each specimen slice were captured and analyzed with Image-Pro Plus-6.0.

For immunofluorescence staining, deparaffinized sections of 2W tissue specimen were first incubated with anti-CD4 and anti-CD68 mouse polyclonal antibody respectively overnight at 4 °C, and then treated with FITC-conjugated anti-mouse IgG antibodies for CD4 and TRITC-conjugated anti-mouse IgG antibodies for CD68 for 120 minutes at 37 °C. All sections were counterstained with Hoechst 33342 dye for 30 seconds at room temperature. Deparaffinized sections of 4W were incubated respectively with anti-GFP, anti-CD31 and anti-CD45 mouse polyclonal antibody as primary antibody and, FITC-conjugated anti-mouse IgG for GFP along with TRITC-conjugated anti-mouse IgG antibodies for CD31 and CD45 as secondary antibodies. Staining procedure was like the above. All primary antibodies were bought from Abcam, USA and used at concentration of 1/200. The data was then collected by a laser confocal microscopy system (Olympus, Japan). 6 fields randomly selected under 40 × visual were subjected to target cell counting and quantification.

### RT-PCR analysis

About 200 mg newly-formed tissue was separated at the central part from wound bed tissue specimen of 2W and 4W, and subjected to tissue homogenizer in Trizol Reagent (Invitrogen) according to the manufacturer’s standard instructions, until no solid tissue left. Total RNA extraction, reverse transcription of mRNA, RT-PCR reaction and analysis method were the same to above.

To detect and compare the inflammatory infiltration of different groups and their variation with time point change, RT-PCR was conducted on several inflammatory concerned cytokines’ Such cytokines included IL-1β and TNF-α at 2W. INOS, as an essential T cell suppression gene, was examined in 2W specimen to reflect MSC immune-regulation capability *in vivo*. Collage I was examined in 4W specimen to confirm histological and immunofluorescence staining. Target genes and primers were presented in Table 1. β-actin was used as internal control and quantified in parallel with target genes.

### Statistical analysis

All data were expressed as means ± SD. Unpaired Student’s t-test was performed to compare two groups. One-way analysis of variance was used for multiple group comparisons followed by Tukey post-test comparing the subgroups. All statistical analysis was performed by SPSS software version 16.0 (SPSS, Chicago, IL). *P* value less than 0.05 was considered as statistical significant.

## Additional Information

**How to cite this article**: An, Y. *et al.* Bone marrow mesenchymal stem cell aggregate: an optimal cell therapy for full-layer cutaneous wound vascularization and regeneration. *Sci. Rep.* **5**, 17036; doi: 10.1038/srep17036 (2015).

## Supplementary Material

Supplementary Information

## Figures and Tables

**Table 1:** The primer sequence for real time PCR.

Gene	Primer sequence
Col-1	F: 5′-CTGACTGGAAGAGCGGAGAG-3′
	R: 5′-TGGGGAACACACAGGTCTGA-3′
TGF-β	F: 5′-CTGCTGACCCCCACTGATAC-3′
	R: 5′-AGCCCTGTATTCCGTCTCCT-3′
VEGFα	F: 5′-GCCTTGTTCAGAGCGGAGAA-3′
	R: 5′-CCTTGGCTTGTCACATCTGC-3′
TNF-α	F: 5′-ATGGGCTCCCTCTCATCAGT-3′
	R: 5′-GCTTGGTGGTTTGCTACGAC-3′
IL-1β	F: 5′-ATCTTTGAAGAAGAGCCCGTCC-3′
	R: 5′-AGCTTTCAGCTCACATGGGT-3′
