# Supplementary material for: Bone marrow mesenchymal stem cell aggregate: an optimal cell therapy for full-layer cutaneous wound vascularization and regeneration
Source: Sci Rep. 2015 Nov 23;5:17036. doi: 10.1038/srep17036 (PMC4655471; doi:10.1038/srep17036)
Supplement: Supplementary Information [file srep17036-s1.pdf]

Bone marrow mesenchymal stem cell aggregate: an optimal cell therapy for full-layer cutaneous wound vascularization and regeneration

Yulin An[a,b,c,1], Wei wei[a,b,d,1], Huan Jing[a,b,1], Leiguo Ming[a,b], Shiyu Liu[a,b], Yan Jin[a,b,*]

[a] *State Key Laboratory of Military Stomatology, Center of Tissue Engineering, School of Stomatology, The Fourth Military Medical University, No.145 Changlexi Road, Xi'an, Shaanxi 710032, China.*

[b] *Research and Development Center for Tissue Engineering, Fourth Military Medical University, No.145 Changlexi Road, Xi'an, Shaanxi 710032, China.*

[c] *Zhen Jiang Entry-Exit Inspection And Quarantine Bureau, No. 84 Dongwu Road, Zhen Jiang, Jiang Su 212000, China.*

[d] *State Key Laboratory of Military Stomatology, Department of Periodontology, School of Stomatology, The Fourth Military Medical University, No.145 Changlexi Road, Xi'an 710032, China.*

[*] *Corresponding author. Department of Oral Histology and Pathology, School of Stomatology, Fourth Military Medical University, No.145 Changlexi Road, Xi'an 710032, Shaanxi, China. Tel.: +86 029 8477 6471; fax: +86 029 8321 8039*

E-mail addresses:yanjinfmmu@vip.sina.com

[1] *Shared first co-authorship as three authors contributed equally to this work.*

Supplementary figures:

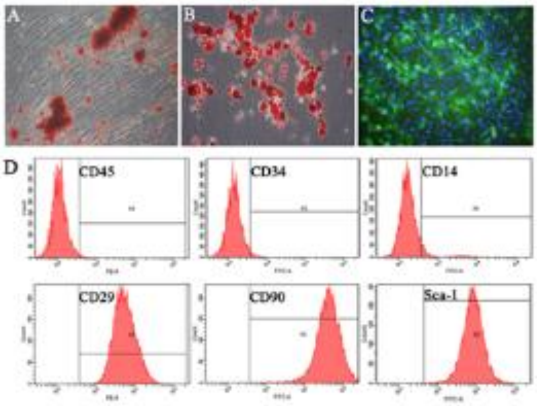

Supplementary Fig. 1

Supplementary Fig. 1: Isolation and identification of GFP$^+$BMMSC.

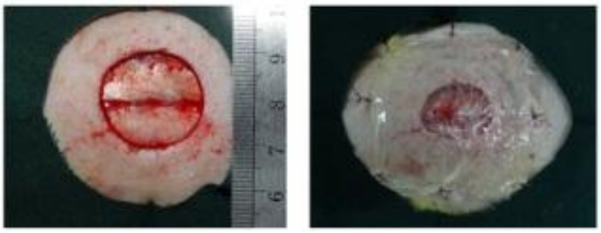

Supplementary Fig. 2

Supplementary Fig. 2: A 2cm diameter full thickness cutaneous wound was made on the top half of the rat back right across the midline of dorsum. A monolayer air permeating wound dressing (Tegaderm film1624, 3M, USA) was applied on SIS membrane after transplantation.

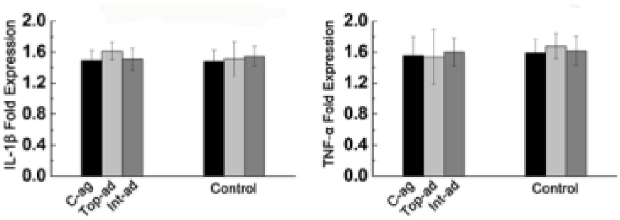

Supplementary Fig. 2

Supplementary Fig. 3: Expression of inflammatory cytokine IL-1β and TNF-α was similar among groups at 1-week post-operation.
